# Health Sciences Interprofessional Collaborative: A Perspective on Migration, COVID-19, and the Impact on Indigenous Communities

**DOI:** 10.3389/fsoc.2021.618107

**Published:** 2021-05-31

**Authors:** Anna Landau, Brenda Sanchez, Lisa Kiser, Jill De Zapien, Elizabeth Hall-Lipsy, Diego Pina Lopez, Maia Ingram, Josefina Ahumada

**Affiliations:** ^1^ University of Arizona, Tucson, AZ, United States; ^2^ Arizona State University, Tempe, AZ, United States

**Keywords:** indigenous, migration, asylum, border health, interprofessional

## Abstract

At the United States-Mexico border, the impacts of immigration policy are dynamic with political, humanitarian, and health outcomes. This article highlights the experiences at the Casa Alitas migrant shelter in Tucson, Arizona. Casa Alitas aims to meet the needs of the im/migrants it serves, including the unique needs of indigenous asylum-seekers from Central America. We highlight the importance of community-based humanitarian response to support asylum-seekers in a way that acknowledges our shared humanity and implements specific approaches (e.g., language justice and trauma informed care). The effort at Casa Alitas is unique because in addition to other partnerships, Casa Alitas established an interprofessional collaboration between the University of Arizona Health Sciences Colleges and the Arizona State University School of Social Work. The interprofessional collaboration encourages mutual education amongst our professions and the use of our extended networks to meet the needs of im/migrants and asylum seekers in our community and the United States. We recommend the development of best practices in asylum health care, the importance of creating border-wide networks to build on local resources, and highlight the importance of exposing future health practitioners to trauma informed and culturally and linguistically appropriate care.

## Introduction

The number of individuals seeking asylum in the United States skyrocketed over the past 2 years, increasing from 55,584 credible fear claims in 2017 to 146,660 in 2019 (U.S. Customs and Border Protection, 2020). During that same period, over 20,000 individuals came through the Casa Alitas Welcome Center, a shelter serving asylum seekers in Tucson, Arizona. These steep increases are a direct result of historic policy changes implemented under the Trump administration. The interaction of these policies with deteriorating socio-economic, political and climate conditions in Central America have served to exacerbate this migration trend. Indigenous populations from the Northern Triangle (Guatemala, El Salvador and Honduras) are disproportionately displaced by these external factors and have increased their migration in recent years (National Immigration Forum, 2019b; Meyer, Peter and Maureen Taft Morales, 2019). Among asylum-seekers at Casa Alitas, approximately one in every six identified as indigenous, and this is likely an undercount of the true numbers (Ama Consultants, 2020).

Casa Alitas is part of a network of migrant shelters and humanitarian aid groups on both sides of the United States-Mexico border working to meet the needs of im/migrants and asylum-seekers and serve as a counterpoint to the harsh shifts in immigration policy, enforcement, and political messaging over the past several years (Associated Press, 2019; Foster-Frau, 2019; Neri, 2019; Stone 2019; Prendergrast, 2020).

This article will address the response to this humanitarian crisis from the perspective of an interprofessional team from the University of Arizona Health Sciences and Arizona State University based at Casa Alitas. We will focus on our experiences and efforts to address the healthcare needs of asylum-seekers, particularly indigenous, in our community, beginning with the increase of asylum-seekers in 2018 and examining the repercussions associated with the COVID-19 pandemic of 2020. This work was done with the intent to provide trauma-informed care focusing on language justice and cultural humility. Based on our experience we offer an account of one community’s response and potential practice recommendations to build a comprehensive, collaborative approach to the care of asylum seekers.

Internationally, we recognize the status of both refugees and asylum-seekers. Refugees, however are afforded both visas and certain health and humanitarian aid benefits while asylum-seekers are in a more tenuous position. In the United States, asylum-seekers live with humanitarian parole, a vulnerable status where they are in constant threat of being detained, and are without explicit access to medical care (Frelick, 2005; Siskin, 2009; American Civil Liberties Union, 2020). In order to address the complex nature of the factors influencing the health of migrant families, a multilateral and interprofessional approach is needed to provide holistic and comprehensive care.

This need became apparent during the response to the rapid influx of migrants into the Tucson community in fall 2018. Faculty and students from the individual health sciences colleges at the University of Arizona (Public Health, Nursing, Pharmacy, and Medicine) recognized that each college was separately mobilizing a response to the urgent needs of the arriving asylum-seekers, and that it would be more effective and serve everyone better to collectively coordinate and build on each college’s work and expertize.

We want to recognize that we are not the center of this experience. We respect the experiences, decisions, and journey of the people we serve and recognize that our ethical duty extends beyond service to ameliorating harmful immigration policies in the United States.

## Impact of Asylum Policies on the United States-Mexico Border

Asylum can be requested from within the United States or at a port of entry (National Immigration Forum, 2019a). At the United States-Mexico border, individuals seeking asylum can also cross into the United States outside of a formal point of entry and present to Border Patrol agents (Miroff, 2018). Prior to 2018, following a credible fear interview, asylum-seekers could be subject to civil detention or released to sponsors in the United States (National Immigration Forum, 2019a). Casa Alitas originated out of a need to support the individuals who were reuniting with sponsors in the United States.

Changes to immigration policy implemented after 2018 created new risks for asylum seekers. These changes included metering, Migrant Protection Protocols (MPP)/“Remain in Mexico”, the asylum transit ban, and the Prompt Asylum Case Review (PACR) program. The MPP policy required asylum-seekers to remain in, or return to, Mexico for the duration of their asylum proceedings (American Immigration Council, 2020a; American Immigration Council, 2020b), and expelled over 60,000 migrants including at least 16,000 children and 500 infants. (Miller et al., 2020). The informal refugee camps for asylum-seekers in Mexico often lack access to clean water, food, safe places to sleep, bathrooms and other essential infrastructure including very limited health services (Miller et al., 2020). This contributes to the spread of illnesses such as COVID-19 and can increase the risk of violence (Human Rights Watch, 2020). Asylum-seekers in these camps reported over 816 incidents of rape, kidnapping, torture and other attacks in 2019 alone (Human Rights Watch, 2020).

Indigenous im/migrants from Central America are especially vulnerable as they are more likely to speak an indigenous language and have limited Spanish. This poses a challenge to navigating the camp environment, accessing health resources, engaging in the limited legal assistance available, and navigating the asylum hearing process. Additionally, members of indigenous cultures are at higher risk of persecution in Mexico (Ahtone, 2018; Long and Sawyer, 2019).

The conditions in the refugee camps under MPP were exacerbated with the onset of the COVID-19 pandemic. In early 2020, the Trump administration effectively closed the United States-Mexico border to asylum seekers, and allowed for the immediate expulsion of noncitizens, including children, arriving at the border (Miller et al., 2020). The administration has since extended this policy indefinitely although the decision has been criticized by public health professionals and was opposed by officials at the Centers for disease Control (Alvarez, 2020; Centers for Disease Control and Prevention, 2020; Columbia University Mailman School of Public Health, 2020; Guttentag and Bertozzi, 2020). This policy led to the removal of 204,000 individuals between April and October 2020 and worsened the already dangerous conditions in the make-shift camps (Miller et al., 2020; Montoya-Galvez, 2020a). Journalists have reported that CBP used the policy to justify the expulsion of United States infants without their United States citizenship paperwork (Srikrishan, 2020; De La Hoz, 2021). Due to COVID-19, many immigration courts have closed and MPP proceedings were halted. These delays prolong stays in refugee camps. The Trump administration also continued to deport immigrants, with numerous deportees testing positive for COVID-19 immediately on arrival in their home countries (Mohammed et al., 2020). At one point, at least 23% of cases in Guatemala could be traced to returned detainees (Montoya-Galvez, 2020b). Indigenous populations are disproportionately impacted by COVID-19, and the pandemic has exacerbated racism and stigma toward these groups, blaming them for higher rates of infection (Pan American Health Organization, 2020).

## Casa Alitas

Since 2014, Casa Alitas has provided food, health care, short-term housing, clean clothing, and travel support for asylum-seekers. Partnership with community organizations and with local jurisdictions have supported the shelter’s services. The 2018 increase in asylum seekers created a renewed imperative to provide equitable and culturally sensitive humanitarian support. In the spring of 2019, the shelter served over 400 individuals at any one time, with more than 200 asylum-seekers arriving daily. In response, Casa Alitas partnered with secular and religious organizations to open pop-up shelters in the community. This network included hundreds of individuals from the Tucson community volunteering their time and expertise.

The asylum-seekers served by Casa Alitas represent vulnerable groups, including many indigenous people from Central America. Prior to the COVID-19 pandemic, nearly all were families–usually a parent with children, and the remainder were single adults with health conditions requiring significant attention which precluded them from remaining in detention due to the documented substandard medical care and dangerous health conditions in civil immigration detention facilities (Siskin, 2009; Long and Meng, 2017). Under the Trump Administrations’ use of MPP and other policies to shut-down the asylum process, Casa Alitas experienced a decrease in activity toward the end of 2019. However, with the COVID-19 pandemic, Casa Alitas has renewed services, meeting a need for support and care for im/migrants who are released from long-term detention facilities or are exempt from the MPP due to medical conditions such as pregnancy, end-stage renal disease, diabetes or other respiratory diseases with higher-risk for COVID-19.

In order to most effectively serve these vulnerable people and ensure equitable care, Casa Alitas volunteers work to identify individuals needing specialized attention, specifically: those separated from their families, indigenous language speakers in need of interpretation services, or those with severe or immediate health needs.

### Factors Impacting Indigenous Asylum-Seekers at Casa Alitas

Mirroring national im/migration trends, Casa Alitas has observed a change in demographics of asylum-seekers, with increases in those arriving from indigenous communities in Central America. Program records indicate that 60% of those served were from Guatemala. Within Guatemala, there are over 20 indigenous languages and dialects, and 21% of Casa Alitas guests reported speaking an indigenous language as their primary language. The most common self-identified indigenous languages spoken by migrants at the shelter were: Mam (42%), Q’eqchi’ (14%), K’iche (10%), and Q’anjob’al (10%).

These language barriers create vulnerabilities across multiple settings including within the informal camps in Mexico, where, as mentioned indigenous experience higher incidence of persecution, and within the United States legal system where the inability to offer adequate interpretation jeopardizes asylum cases (Jawetz and Scott, 2019; Medina, 2019; Nolan, 2019).

Indigenous groups in Central America, much like those in the United States, faced persistent barriers (e.g., discrimination in language, education, and health access) and historical trauma related to violence and displacement. During the initial arrival of asylum-seekers at the Casa Alitas shelter, many individuals did not volunteer their indigenous primary language or their immediate health concerns. This resulted in delays in communication and care. Additionally, the self-identified reporting of indigenous language described above is likely an underestimate due to this hesitancy to volunteer such information. Casa Alitas recognized this phenomenon early on and worked closely with indigenous language specialists to address these delays and mitigate the language barriers contributing to the perpetuation of health disparities.

In response to the numbers of Guatemalans who have come through the shelter, and the desire to provide equitable assistance, Casa Alitas established a relationship with the Guatemalan Consulate. This relationship expedited the process of arranging legal, travel, and language services and reuniting family members. Casa Alitas also worked to address the issue of language access, by creating and maintaining a network of peer interpreters. These are individuals who are fluent in an indigenous language as well as either Spanish or English, who were willing to serve as on-call phone or in-person interpreters. While many lacked formal training; they had passed through the shelter themselves and their dedication bridged the gaps for those following in their footsteps. The peer interpreters demonstrated compassion and commitment that continues to benefit those in their direct service and the broader community. Additionally, the repeated need to use appropriate and culturally sensitive interpreters has increased local provider and volunteer awareness, along with improving cultural humility in healthcare and social services practice.

Despite efforts to mitigate the substantial barriers that asylum-seekers faced, it is clear that many experienced additional challenges in their journeys. These included persistent language and sociocultural barriers. Indeed, this vulnerable population may often seem invisible in communities unprepared to deliver the support that they require. These individuals and families often experience continued persecution and fear of the immigration processes. While these barriers exist for all migrants, they are especially pronounced for indigenous groups.

### Medical and Interprofessional Response at Alitas

Individuals seeking asylum, im/migrating, or arriving as refugees often present with histories of trauma incurred pre-departure, during their migration journey, after arrival and during detention, and while assimilating into their new communities (The Center for Victims of Torture, 2005). This is certainly true for those arriving at Casa Alitas. After making the difficult decision to leave their home countries, many experience arduous trips through Central America and Mexico. They may be persecuted or extorted by the cartels, and experience further trauma upon arrival at the United States-Mexico border.

At the United States-Mexico border many waited days, if not months, to be allowed to seek asylum. Others crossed through treacherous routes in the desert, only to then be detained and held in “hieleras” or “ice-boxes” by CBP (Garcia Bochenek, 2018; National Immigration Forum, 2019a; National Immigration Law Center, 2020). This mistreatment at the hands of the United States Government and others are efforts to diminish dignity. Asylum-seekers arrive at the shelter with ID bracelets from CBP but without their shoelaces, hair ties, necessary medicines, and sometimes missing personal belongings. Casa Alitas attempts to restore some of that dignity.

A team of doctors, NPs, PAs, nurses, pharmacists, public health professionals and social workers formed to address the trauma and health needs of the guests arriving at the shelter. As mentioned, there is precedent for formally integrating refugees into our health system and society, with pre-departure screenings, post-arrival clinic visits, and agencies with dedicated staff to accompany individuals through the arrival process. There is no parallel system for those seeking asylum. The experiences of those arriving at the shelter demonstrated the need for a community-based and interprofessional response to meet their health needs. The Casa Alitas healthcare team and its interprofessional composition have, as a result, formed an external support system and network that has served as a bridge to the existing infrastructure both in Tucson and in the communities where these individuals settle. This has included partnerships with the local public health department and universities to provide more comprehensive services including flu vaccines, well-child visits, and varicella and flu treatment and prophylaxis.

At Casa Alitas, the medical team screens all asylum-seekers on arrival at the shelter. They are assessed for urgent, serious, or infectious conditions and triaged appropriately. Following the initial screening, asylum-seekers have access to medical care on an as-needed basis, with an on-call provider available 24 h a day both to triage and address immediate health needs and accompany those who need higher levels of care at local clinics or hospitals. Nearly all presented with some level of dehydration and malnutrition. They reported gastrointestinal symptoms from poor quality food and hesitancy to drink while in detention exacerbating dehydration and food scarcity from their journeys. Internal project records show that pre-COVID, one in ten reported severe or acute medical conditions on arrival such as diabetes, hypertension, or even cancer or HIV, and approximately 4% were pregnant. After MPP and COVID-19, fewer guests arrived, but those arriving required significantly increased medical attention. Many of those released from detention facilities were directly exposed to COVID-19, with some testing positive, and requiring hospitalization. Many others had pre-existing chronic conditions that put them in high-risk categories and needed higher levels of medical care.

Of those coming directly from the border, some of those with serious conditions were treated during their brief detentions by ICE or CBP. Many others, however, reported that their medications were confiscated during their detention. Others reported recent surgeries or stays at local hospitals, but often arrived without discharge information, medications, or follow-up plans. Due to crowded conditions on the journey and in detention, many arrived with infections such as flu, varicella, scabies, and hand-foot-mouth disease. In the shelter setting, it is important to identify and treat such infections prior to further outbreaks. This became even more essential following the onset of the COVID-19 pandemic. Other asylum-seekers experienced rape or sexual assault during their journey or prior to leaving their home countries. Still others had visible signs of Post-Traumatic Stress Disorder (PTSD) and manifestations of torture that occurred during or prior to their journeys.

In addition to direct medical care, there was a need for a more comprehensive and interprofessional approach to address the complex needs of the migrants. To this end, we formed a standing interprofessional working group of faculty and staff from University of Arizona Health Science colleges and the Arizona State University School of Social Work, This group has partnered with community organizations and has allowed us to be dynamic and adaptable to the unfolding needs of families welcomed at the shelter. The group lends academic support to the ongoing crisis and integrates students into the volunteer teams. In partnership with SEAHEC (Southeast Arizona Area Health Education Center), we created an interprofessional service-learning course, Migration Interprofessional Leading to Action and Growth (MILAGRO), to provide first-hand experience with the realities of humanitarian care, and expertize in building sustainable, community-engaged responses. Students work in interprofessional teams to learn about the root causes and impacts of migration and the realities and challenges migrants and asylum-seekers face, preparing students for a more culturally informed, interprofessional approach to their future practices. Projects have ranged from creating videos on how to navigate travel across the United States using the bus system, to in-person instruction on recovering from dehydration and malnutrition. This model of an academic-community partnership, based in interprofessionalism, is one that we want to share with our communities, nationally and internationally, as they seek to be responsive to the urgent health issues in their own communities and nations.

## Discussion

With this paper, we aimed to share our experiences responding to the humanitarian crisis caused by punitive immigration policies with a focus on addressing the needs of indigenous people who are migrating to the United States. As an interprofessional group, we have witnessed the devastating effects of policy changes on the individuals seeking asylum and, on their families, and communities. The interprofessional nature of our partnership allowed us to view the complexity of the issues facing asylum-seekers from a diversity of perspectives and develop a robust and evolving response as aspects of the crisis changed in response to COVID-19. Sharing information within our group and with the participating students has led to increased knowledge and improved practices in the areas of cultural humility, language justice, trauma informed care, and healthcare.

The medical needs of asylum-seekers that we observed among those released from detention underscore the need for the development of best practices on integrating asylum healthcare into community-based health care. Our experiences caring for im/migrants confirm the extensive reporting on the inadequacy of care in CBP and civil detention settings. Humanitarian workers must be prepared to assess and respond to chronic conditions that have been exacerbated by the journey. Given the substantial expertize and medical resources needed to provide a comprehensive response in a given community, further development of networks across communities and binationally is warranted.

Lack of access to adequate interpretation services perpetuates the inequalities faced by all im/migrants. Indigenous asylum-seekers face language and cultural barriers that compound these challenging situations. While it is, or should be, protocol for individuals to be offered formal interpretation services both in medical and legal settings, we found that the reality is more complicated. Many indigenous im/migrants hesitate to disclose their language preferences for fear of discrimination. In the best of scenarios it is still difficult to obtain accurate interpretation or translation of resources into the dialects of numerous indigenous languages (Gentry, 2015). Numerous national organizations are working toward addressing linguistic justice and addressing adequate interpretation services for indigenous migrants in legal and medical settings. Supporting and connecting with these organizations and using technology across communities receiving asylum-seekers could aid this effort. Further, we recommend the creation of a cadre of paid professional interpreters working in legal and health care settings to ensure linguistic justice. Awareness of the language and other barriers faced by indigenous im/migrants and asylum seekers is an important starting point but it is insufficient to meet the standards of ethical care.

While this article focuses on the experiences and lessons learned from one community shelter, further studies and attention are needed to understand how im/migrants, and particularly indigenous migrants, are integrating into their destination communities, and how to better support them and their communities in this process. In Tucson, the community came together, with hundreds of volunteers working with community organizations like Casas Alitas and complemented by partnerships with local governmental agencies, other non-profits, and the resources and infrastructure of an academic partnership. This dynamic partnership has helped to address the crisis created by immigration policy focused on restriction and enforcement and that refuses to engage or address the human consequences of such policies.

Our experience recommends the creation of interprofessional networks for humanitarian responses in order to ensure ongoing sharing of experiences, failures and successes, and to identify strategies for improvement. Additionally, we have an opportunity and obligation to train future medical, social, public health and legal providers in a more inclusive, interprofessional, comprehensive, compassionate, and culturally humble manner. This will ensure trauma-informed care that is team-based and culturally and linguistically appropriate.

## Data Availability

The raw data supporting the conclusion of this article will be made available by the authors, without undue reservation.
